# [Corrigendum] Alcohol extracts from *Anemone flaccida* Fr. Schmidt treat rheumatoid arthritis via inhibition of synovial hyperplasia and angiogenesis

**DOI:** 10.3892/mmr.2024.13302

**Published:** 2024-08-09

**Authors:** Qi Rao, Xin Zhao, Fenghua Wu, Xiaohong Guo, Yundan Xu, He Yu, Dayong Cai, Gang Zhao

Mol Med Rep 27: 88, 2023; DOI: 10.3892/mmr.2023.12975

Subsequently to the publication of the above paper, the authors drew to the attention of the Editorial Office that they had assembled the data shown for the cell migration assay experiments in Fig. 4F (on p. 8), incorrectly; essentially, the ‘Control’ data panel had inadvertently been copied across for the ‘10 μg/ml’ data panel.

The revised version of Fig. 4, showing the correct data panel for the ‘10 μg/ml’ experiment in Fig. 4F, is shown on the next page. Note that the replacement of the erroneous data does not affect either the results or the conclusions reported in this paper, and all the authors agree to the publication of this Corrigendum. The authors are grateful to the Editor of *Molecular Medicine Reports* for granting them this opportunity to publish a Corrigendum, and apologize to the readership for any inconvenience caused.

## Figures and Tables

**Figure 4.**
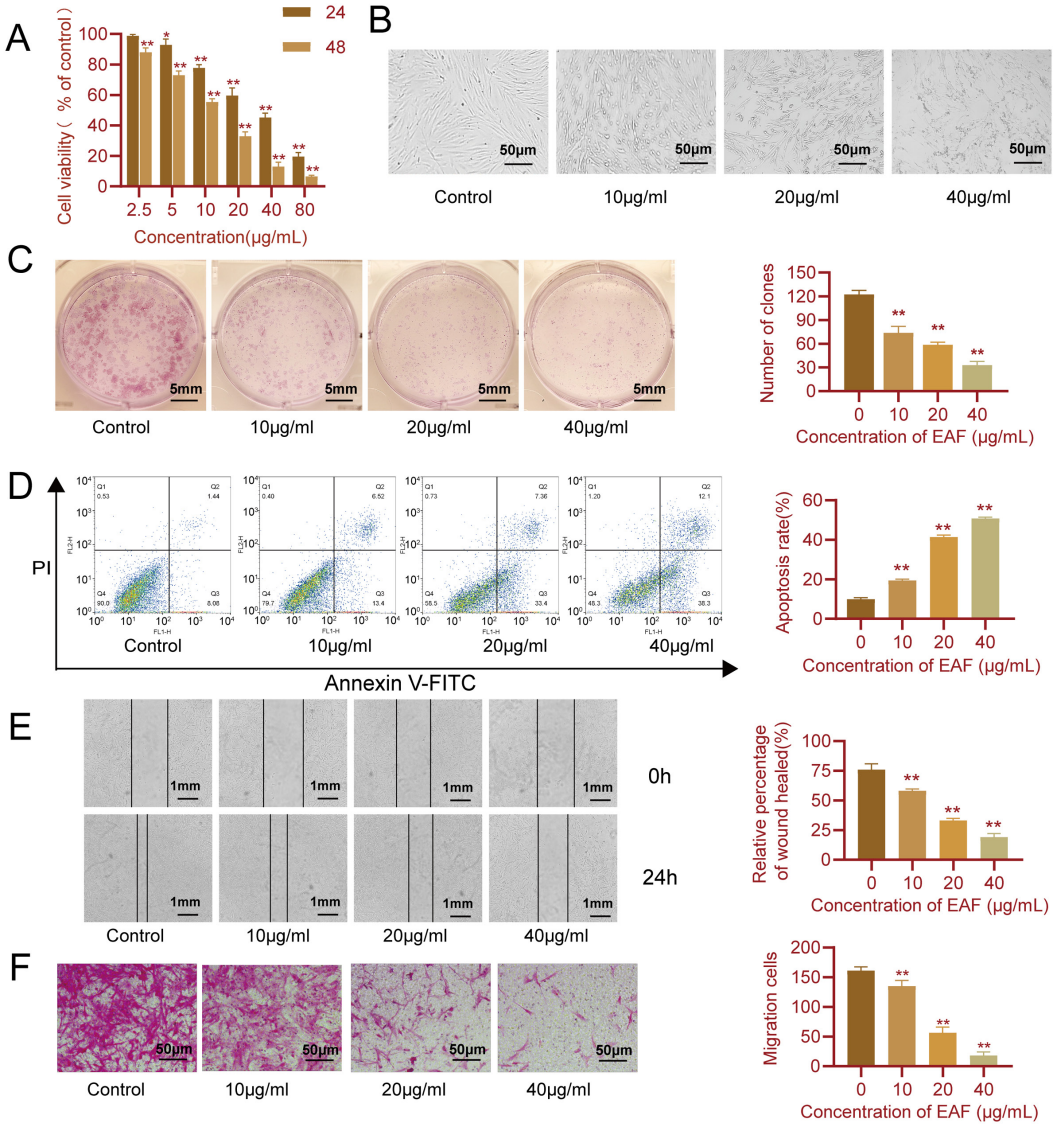
EAF inhibits the proliferation and migration of synovial cells and promotes apoptosis. (A) Viability of HFLS-RAs detected using a Cell Counting Kit-8 assay following treatment with EAF for 24 or 48 h. (B) Images of HFLS-RAs following treatment with different concentrations of EAF for 48 h. (C) Colony formation of HFLS-RAs following treatment with EAF. (D) Apoptosis of HFLS-RAs following treatment with different concentrations of EAF for 24 h was detected using flow cytometry. (E) Wound healing assay was used to detect the migration of HFLS-RAs following treatment with different concentrations of EAF. (F) Images of migrated HFLS-RAs following treatment with different concentrations of EAF. Data are expressed as the mean ± standard deviation. Experiments were carried out at least three times. *P<0.05, **P<0.01 vs. control group. EAF, ethanol extract of *Anemone flaccida* Fr. Schmidt; HFLS-RAs, human fibroblast-like synoviocytes-RA.

